# Three Adult Cases of ADHD With Comorbid Tic Symptoms Successfully Treated With Guanfacine Monotherapy

**DOI:** 10.1002/npr2.70167

**Published:** 2026-09-07

**Authors:** Koji Tada, Teruo Tada, Takashi Tsuboi, Koichiro Watanabe

**Affiliations:** ^1^ Jimbocho Mental Health Clinic Tokyo Japan; ^2^ Department of Neuropsychiatry Kyorin University School of Medicine Tokyo Japan

**Keywords:** adult ADHD, Guanfacine, monotherapy, tic disorder

## Abstract

Tic disorders typically begin in childhood and often improve by adulthood, but some individuals continue to experience tic symptoms into adult life. Tic disorders frequently co‐occur with Attention‐deficit/hyperactivity disorder (ADHD), and in such cases α2‐adrenergic agonists are recommended as a treatment as they can address both conditions. However, evidence in adults with ADHD and comorbid tic disorders is limited, as most studies have focused on pediatric populations. Here, we present three adult male patients with childhood‐onset ADHD and chronic tic symptoms who showed a rapid and significant improvement in tics with low‐dose extended‐release guanfacine (GXR) monotherapy. These cases suggest that GXR can effectively treat both ADHD and tic symptoms in adults—a finding that has not been well documented in prior adult studies.

## Abstract 2

Guanfacine extended‐release can be an effective monotherapy for adults with ADHD and comorbid tic disorders. A low dose of guanfacine was sufficient to produce a rapid improvement in tic symptoms in each patient before improving ADHD symptoms.

## Graphical abstract

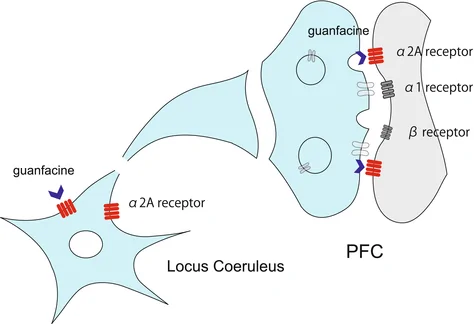

## 1 Introduction

Tic disorders are characterized by involuntary, rapid motor movements or vocalizations. Motor tics include behaviors such as blinking, shoulder shrugging, or facial grimacing, while vocal tics encompass sounds like coughing, sniffing, or grunting. In its most severe form, Tourette syndrome (defined by multiple motor tics and at least one vocal tic persisting for over a year) often involves coexisting neuropsychiatric conditions and tends to present with greater severity and comorbidity than simple chronic tics [1]. Most tic symptoms improve or remit by adulthood, and it is uncommon for an adult to present with tics as the primary complaint. When tics do persist into adulthood, they are frequently overshadowed by co‐occurring conditions such as ADHD or obsessive‐compulsive disorder (OCD) [2].

ADHD is among the most common comorbidities in individuals with tic disorders. For patients with co‐occurring ADHD and tics, treatment can be challenging. CNS stimulant—first‐line treatments for ADHD—can occasionally exacerbate tic symptoms, so alternative therapies are often considered [3]. Alpha2‐adrenergic agonists like clonidine or guanfacine are recommended in such cases because they can reduce ADHD symptoms without provoking tics, and in fact have therapeutic effects on tics themselves. Indeed, the 2019 American Academy of Neurology (AAN) guidelines for tics and Tourette syndrome (when comorbid with ADHD) recommend clonidine or guanfacine as first‐line pharmacotherapy [4], and similar guidance is echoed in European clinical guidelines [5]. Notably, clonidine has traditionally been used, but guanfacine—being more selective for α2A receptors and causing fewer hypotensive side effects—has been increasingly used in recent years.

However, although the efficacy of α2 agonists in children with ADHD and tics is supported by several studies, data regarding their effectiveness in adults remain limited. The first author previously reported a single adult case treated with guanfacine in Japanese [6]. The present report adds two additional cases and provides further evidence supporting the potential efficacy of guanfacine monotherapy in adult patients with ADHD and persistent tic symptoms. Here, we report three such adult cases in which low‐dose guanfacine extended‐release (GXR) alone led to a clear and relatively rapid improvement in tic symptoms. All three patients had childhood‐onset ADHD with chronic motor and/or vocal tics, yet none had received psychiatric treatment until adulthood. Written informed consent was obtained from all patients, and all identifying information has been removed to protect their privacy.

## 2 Case Presentations

### 2.1 Case 1

A 40‐year‐old man with no prior psychiatric history presented to our clinic seeking evaluation for lifelong problems with attention and involuntary tics. After graduating from high school, he has been employed in the security staff sector.

ADHD history: Since early childhood he had been inattentive and forgetful, often losing items and struggling to follow instructions. As a student, he was frequently described as restless and had difficulty remaining seated, often fidgeting with objects (such as pencils or other stationery) during the class. These attention deficits and hyperactive behaviors persisted into adulthood. At work, he found it challenging to adhere to plans and deadlines, and he remained physically fidgety—swaying in his seat, swinging his legs, and frequently looking around even in meetings when stillness was expected.

Tic history: The patient's motor tics began at approximately 7 years of age, initially manifesting as frequent blinking and quick shoulder jerks that were noticeable to others. The severity of these tics fluctuated over time but never fully resolved. In his late twenties, he also developed vocal tics. He would involuntarily utter an “Ah~” sound, which eventually progressed to involuntary vocalizations of expletives (e.g., shouting “damn it”) immediately after the simple vocal tic. These vocal outbursts were distressing and socially embarrassing for him. Despite the persistence of tic symptoms from childhood onward, he had never sought psychiatric care until then, likely as he had adapted to the situation and his overall impairment was mild. According to the DSM‐5TR [7] criteria, the patient was diagnosed with comorbid Tourette's disorder and ADHD, combined presentation, mild severity.

Baseline assessment: At the time of presentation, all subscales of the Conners' Adult ADHD Rating Scale (CAARS) were elevated (T‐scores > 65), consistent with marked ADHD symptomatology. On mental status examination, he appeared overtly restless, frequently shifting in his chair. His Yale Global Tic Severity Scale (YGTSS) scores at baseline indicated mild‐to‐moderate tic severity (motor tic score 9, vocal tic score 11).

Treatment and outcome: We initiated GXR monotherapy at 1 mg daily. Within 2–3 days of starting treatment, the patient noted a dramatic reduction in his tic symptoms. The involuntary “Ah~” vocalizations ceased entirely, as did the involuntary shouting of expletives. His motor tics, such as excessive blinking and shoulder movements, also diminished markedly. The patient reported feeling markedly calmer overall. A mild residual restlessness remained (he would occasionally tap his foot when sitting), but this was much less pronounced than before. Following four weeks of treatment, the GXR dose was increased to 3 mg daily to address persistent inattention and impulsivity. Two weeks after the dose escalation to 3 mg, CAARS was reassessed and showed measurable improvement in hyperactivity and impulsivity; however, scores remained clinically elevated, exceeding the cutoff value of 65 (Table 1). Despite this, the patient reported only infrequent fidgeting and no longer exhibited overt restlessness during clinical visits. Importantly, his tic symptoms remained well controlled; YGTSS scores obtained two weeks after the increase to 3 mg reflected this stability, with the motor tic score decreasing from 9 to 5 and the vocal tic score resolving from 11 to 0 (Table 2). He tolerated the medication well, without any sedation or other side effects.

**TABLE 1:** Conners' Adult ADHD Rating Scales scores before and after Guanfacine administration.

CAARS subscale	Before treatment		After Guanfacine administration	
	Total score	T score	Total score	T score
**Case 1**				
(A) Inattention/Memory Problems	24	82	24	82
(B) Hyperactivity/Restlessness	26	90	21	81
(C) Impulsivity/Emotional Lability	26	77	26	77
(D) Problems with Self‐Concept	18	90	18	90
(E) DSM‐IV: Inattentive Symptoms	16	90	16	90
(F) DSM‐IV: Hyperactive–Impulsive Symptoms	16	84	12	74
(G) DSM‐IV: ADHD Symptoms Total	32	90	28	84
(H) ADHD Index	25	89	24	87
**Case 2**				
(A) Inattention/Memory Problems	19	68	18	67
(B) Hyperactivity/Restlessness	15	72	14	70
(C) Impulsivity/Emotional Lability	21	79	12	62
(D) Problems with Self‐Concept	15	78	12	70
(E) DSM‐IV: Inattentive Symptoms	15	81	15	81
(F) DSM‐IV: Hyperactive–Impulsive Symptoms	9	73	9	73
(G) DSM‐IV: ADHD Symptoms Total	24	80	24	80
(H) ADHD Index	23	79	21	76
**Case 3**				
(A) Inattention/Memory Problems	31	88	25	78
(B) Hyperactivity/Restlessness	34	90	24	90
(C) Impulsivity/Emotional Lability	26	88	22	81
(D) Problems with Self‐Concept	16	80	15	78
(E) DSM‐IV: Inattentive Symptoms	24	90	18	89
(F) DSM‐IV: Hyperactive–Impulsive Symptoms	25	90	19	90
(G) DSM‐IV: ADHD Symptoms Total	49	90	37	90
(H) ADHD Index	28	88	22	77

**TABLE 2:** Yale Global Tic Severity Scale scores before and after Guanfacine administration.

	Before treatment	After Guanfacine administration
Case 1		
Yale Global Tic Severity Scale		
Motor Tic	9	5
Vocal Tic	11	0
Case 2		
Yale Global Tic Severity Scale		
Motor Tic	10	3
Vocal Tic	0	0
Case 3		
Yale Global Tic Severity Scale		
Motor Tic	9	7
Vocal Tic	10	8

### 2.2 Case 2

A 22‐year‐old man with no past psychiatric history was referred for evaluation of attention difficulties and persistent motor tics. He left university and is currently enrolled in a vocational school.

ADHD history: As a child, he had difficulty sustaining attention, especially during lengthy conversations or tasks. He was easily distracted by background noises and often could not follow through on multi‐step instructions. Teachers and family frequently observed his inability to sit still; he would tap his hands and feet whenever he had to remain seated. In adulthood, these symptoms continued to interfere with his daily functioning. At his job, he struggled with task organization and would procrastinate, having trouble prioritizing his workload. He also remained very sensitive to ambient noise—conversations in crowded environments were hard for him to follow as he would be distracted by irrelevant sounds. His tendency to lose focus led to frequent small mistakes at work, drawing criticism from his supervisors.

Tic history: At around 6 years old, the patient felt an uncomfortable sensation in his throat that he could only relieve by making a soft grunting sound (“uh~”). This behavior was recognized as a simple vocal tic. He also began exhibiting a motor tic consisting of forceful blinking. These tics fluctuated over time; notably, the vocal tic subsided as he grew older and was no longer present by his late teenage years, but some motor tics persisted into adulthood. In the year prior to presentation, he developed a new habit of tensing his abdominal muscles in response to a nonspecific internal discomfort, sometimes without conscious awareness. Additionally, he started rapidly bending his little finger repetitively—a movement that would occasionally become forceful enough to cause him pain. Friends and family had commented on these behaviors, which helped prompt him to seek help. According to the DSM‐5TR criteria, the patient was diagnosed with comorbid persistent motor or vocal tic disorder and ADHD, combined presentation, mild severity.

Baseline assessment: On initial evaluation, he met criteria for ADHD and chronic motor tic disorder. His CAARS assessment showed elevated T‐scores across all subscales, indicating clinically relevant ADHD symptoms in multiple domains. During the interview, he appeared mildly restless (frequently shifting posture and fidgeting with his hands). At baseline, his YGTSS motor tic severity score was 10 (reflecting moderate motor tics), while his vocal tic score was 0 (consistent with the absence of any active vocal tics in adulthood).

Treatment and outcome: We started GXR monotherapy at 1 mg daily. Within the first week, there was a clear improvement in his tic symptoms. The frequency of his rapid eye‐blinking and finger movement tics decreased substantially. He also noted that the urge to tense his abdominal muscles was much less frequent. On the YGTSS, his motor tic score improved from 10 to 3 after beginning GXR (Table 2). Despite this progress in tic control, his ADHD symptoms showed minimal change at the 1 mg dose. He continued to experience significant distractibility, poor concentration, and restlessness. After one month, the GXR dose was increased to 2 mg daily to better address his ADHD symptoms. Two weeks after the dose escalation to 2 mg, CAARS was evaluated and revealed modest improvements in the Impulsivity/Emotional Lability subscale and the Problems with Self‐Concept subscale (Table 1). However, other ADHD symptoms persisted—he was still easily distractible, had trouble sustaining attention in lengthy discussions, and remained somewhat restless and disorganized (Table 1). There was no further change in tic symptoms at the higher dose (which remained minimal after the initial improvement). Overall, he tolerated GXR without any complaints of side effects, and his remaining ADHD symptoms were managed with behavioral strategies.

### 2.3 Case 3

A 27‐year‐old man with lifelong ADHD symptoms and a history of tics presented for treatment after losing another job due to inattentiveness.

ADHD history: In childhood, he was extremely active and inattentive. He could not stay seated for long and often wandered around the classroom or home when he was supposed to be doing schoolwork. He was also very forgetful and had difficulty organizing tasks or keeping track of his belongings. His parents and teachers frequently described him as “always on the go” and noted that his living space was usually messy and disordered. As an adult, his attention problems and hyperactivity continued. He often became absorbed in trivial activities while neglecting important responsibilities. This contributed to an unstable employment history; he found it hard to hold a steady job and was unemployed at the time he came to our clinic.

Tic history: The patient's motor and vocal tics began at around age 7. His early tics included rapid head shaking movements, mouth noises, and occasional grunt‐like vocalizations (“uh~”). These tics persisted into adolescence and adulthood, though he generally considered them a minor nuisance and was not personally very distressed by them. Interestingly, he also developed a behavior of suddenly shouting during moments when he recalled distressing memories. He described these vocal outbursts as a way to “snap himself out” of painful recollections; although this behavior had a volitional aspect as a coping mechanism, it had become almost reflexive over time. Friends who spent time with him observed that his various tics occurred frequently, which eventually led him to seek evaluation. According to the DSM‐5TR criteria, the patient was diagnosed with comorbid persistent motor or vocal tic disorder and ADHD, combined presentation, moderate severity.

Baseline assessment: The patient had never previously received psychiatric treatment. At intake, his CAARS scores confirmed significant attention deficits and hyperactivity/impulsivity (all subscale T‐scores were elevated). In the clinical interview, he was notably restless, frequently changing sitting positions and gesturing. His baseline YGTSS scores indicated mild tic severity (motor tic score of 9 and vocal tic score of 10).

Treatment and outcome: GXR monotherapy was initiated at 1 mg daily. After two weeks on this regimen, the patient reported that both his motor and vocal tic symptoms had roughly halved in frequency and intensity. On the YGTSS, his motor tic score improved from 9 to 7, and his vocal tic score improved from 10 to 8 (Table 2). He and his friends noticed that the head jerks, mouth noises, and grunting sounds were less pronounced. However, his habit of shouting in response to intrusive memories did not change with the low‐dose treatment, and his ADHD symptoms remained largely unchanged. Consequently, the GXR dose was increased to 2 mg daily after one month. Two weeks after the dose escalation to 2 mg, CAARS was assessed. Although the scores remained above the clinical cutoff, those for Inattention/Memory Problems and Impulsivity/Emotional Lability showed a reduction (Table 1). The patient reported feeling “calmer in my mind” and found it easier to remain seated without the urge to pace. He was also less easily distracted and noted reduced forgetfulness. Importantly, the gains in tic control achieved at 1 mg were maintained at the higher dose, although the specific vocal behavior tied to unpleasant memories persisted (likely because it was more of a habitual coping action than a true tic). The patient did not report any adverse effects from GXR, and he felt the treatment helped him function better overall.

## 3 Discussion

We have reported three adult cases of ADHD with comorbid tic disorders that were successfully treated with guanfacine extended‐release monotherapy. In all three patients, tic symptoms markedly improved within days of starting a low dose of GXR (1 mg), preceding significant changes in their ADHD symptoms. ADHD symptoms only began to show improvement after the GXR dose was increased to 2 mg or higher. This pattern suggests that the reduction in tics was not merely secondary to better control of ADHD symptoms; rather, guanfacine appears to have a direct suppressive effect on tic symptoms even at a low dose. Guanfacine primarily acts on postsynaptic α2A‐adrenergic receptors and is known to reduce noradrenergic activity, particularly within the prefrontal cortex. This effect may occur even at relatively low doses and could attenuate excessive neural excitability, thereby suppressing the hyperactivity of motor output circuits thought to contribute to tic symptoms. In contrast, improvement of ADHD symptoms may require a greater degree of α2A‐receptor stimulation sufficient to optimize prefrontal cortical network functions, including working memory, inhibitory control, and sustained attention [8].

All three patients had experienced both ADHD and tics since childhood, yet none had sought treatment until adulthood. One reason might be that their symptoms were relatively mild in childhood and did not cause substantial functional impairment until later in life. Indeed, each patient's baseline YGTSS scores fell in the mild‐to‐moderate range, indicating that while the tics were persistent, they were not severely disabling. It is also possible that the patients coped with their own symptoms during youth and only when adult responsibilities increased did the combined burden of ADHD and tics prompt them to seek help.

The co‐occurrence of tic disorders and ADHD is well documented. Epidemiological studies report that 50%–60% of individuals with Tourette syndrome or chronic tics also meet criteria for ADHD [1]. Conversely, having ADHD is associated with an elevated risk of developing tic disorders [9]. When treating patients who have both conditions, clinicians must balance the need to alleviate ADHD symptoms against the potential to aggravate tics. CNS stimulants, such as methylphenidate, lisdexamfetamine, and dexamfetamine, are highly effective for ADHD in general, but it has been reported that they may exacerbate tic symptoms [10]. Although the incidence of stimulant‐induced tic exacerbation is low, in clinical practice, caution should be exercised in these complex cases. In contrast, α2 agonists are often a preferred option when ADHD and tic disorders coexist, as they can ameliorate symptoms of both disorders simultaneously. The 2019 American Academy of Neurology (AAN) guidelines on Tourette syndrome and chronic tics (with comorbid ADHD) give a Level B recommendation for clonidine and guanfacine, supporting their use as first‐line therapies in such situations [4]. Similarly, the 2022 European clinical guidelines note that clonidine and guanfacine have among the most favorable efficacy‐to‐adverse effect ratios in patients with co‐occurring ADHD and tics [5]. In practice, clonidine has been used for decades to treat tics, but guanfacine's greater α2A receptor selectivity and lower risk of causing hypotension or sedation make it an appealing alternative [11].

Our case series adds to the growing body of evidence supporting guanfacine for tic disorders. Guanfacine has demonstrated efficacy in pediatric populations with tics and ADHD. For instance, in a randomized controlled trial of children with Tourette syndrome and ADHD, guanfacine not only improved ADHD symptoms by about 37% compared to placebo, but also led to a significant 31% reduction in tic severity (versus no improvement in tics in the placebo group) [3]. There are also reports of guanfacine monotherapy successfully treating children with comorbid ADHD and Tourette syndrome [12], and of guanfacine used in combination with antipsychotics like blonanserin in refractory pediatric cases [13]. By contrast, systematic studies of guanfacine in adult ADHD have been scarce. A Japanese trial was among the first to establish that guanfacine is effective for core ADHD symptoms in adults (demonstrating significant improvements in ADHD Rating Scale scores compared to placebo) [14]. Yet, prior to the present report, the specific question of whether guanfacine can alleviate tic symptoms in adult ADHD patients had not been examined in the literature. Our cases are therefore notable in showing that GXR monotherapy was associated with a rapid improvement in tics in adult patients, even before their ADHD symptoms were fully addressed.

The mechanism by which guanfacine exerts its effects on tics and ADHD is not completely understood. Guanfacine is thought to strengthen prefrontal cortical regulation of behavior by stimulating post‐synaptic α2A receptors on pyramidal neurons. Activation of these receptors enhances the functional connectivity of prefrontal networks, which can improve attention and executive function [15]. This mechanism might also contribute to tic suppression, as stronger top‐down control from the prefrontal cortex could help inhibit the emergence of tics. Additionally, unlike CNS stimulants, guanfacine does not increase dopaminergic neurotransmission; given that enhanced dopamine signaling can be associated with worsening of tics, guanfacine's lack of direct dopamine release may make it particularly suitable for patients with tic disorders.

There are some limitations to consider in interpreting these cases. As an uncontrolled observation of three patients, we cannot definitively separate the effects of guanfacine from the natural waxing‐and‐waning course of tic disorders or from potential placebo effects. However, the consistency of the response (all three patients experienced clear tic reductions shortly after starting guanfacine, without any subsequent worsening) makes a purely spontaneous improvement less likely. Another limitation is that all mild tics; whether guanfacine monotherapy would be as effective in adults with more severe Tourette syndrome remains an open question. We also note that while tics improved quickly, the ADHD symptom improvements required higher doses and were somewhat variable among the cases, highlighting that optimal dosing may differ for the two symptom domains.

## 4 Conclusion

In summary, these three cases illustrate that guanfacine extended‐release can be an effective monotherapy for adults with ADHD and comorbid tic disorders. A low dose of guanfacine was sufficient to produce a rapid improvement in tic symptoms in each patient, before improving ADHD symptoms. Our observations suggest that guanfacine might be a safe and beneficial first‐line treatment in such cases. Further research, ideally in controlled trials, is warranted to confirm the efficacy of guanfacine for tic reduction in adult ADHD patients and to establish the generalizability of these findings.

## Author Contributions

K.T. conceived the study, collected clinical data, and drafted the manuscript. T. Tada, T. Tsuboi, and K.W. contributed to data interpretation and critical revision of the manuscript. All authors reviewed and approved the final version of the manuscript and agree to be accountable for all aspects of the work.

## Funding

The authors have nothing to report.

## Consent

Written informed consent was obtained from all patients for publication of this case series and any accompanying clinical information.

## Conflicts of Interest

K.T. declares no conflicts of interest. Teruo Tada has received speaker's fees from Dainippon Sumitomo Pharma, Otsuka Pharmaceutical. Takashi Tsuboi has received grants from Japan Society for the Promotion of Science and honorarium from Takeda Pharmaceutical, Otsuka Pharmaceutical, Meiji Seika Pharma, Shionogi Pharma, Yoshitomiyakuhin, Sumitomo Pharma, Kyowa Pharmaceutical, MSD, Nippon Boehringer lngelheim, Mylan EPD, Mitsubishi Tanabe Pharma, Viatris, Mochida Pharmaceutical, Janssen Pharmaceutical, TEIJIN PHARMA, and Lundbeck Japan. K.W. has received manuscript fees or speaker's honoraria from Eisai, Eli Lilly, Janssen Pharmaceutical, Kyowa Pharmaceutical, Lundbeck Japan, Meiji Seika Pharma, Mitsubishi Tanabe Pharma, MSD, Otsuka Pharmaceutical, Pfizer, Shionogi, Sumitomo Pharma, and Takeda Pharmaceutical and received research/grant support from Daiichi Sankyo, Eisai, Meiji Seika Pharma, Mitsubishi Tanabe Pharma, MSD, Otsu‐ka Pharmaceutical, Pfizer, Sumitomo Pharma, and Takeda Pharmaceutical. In addition, K.W. is a consultant of Boehringer Ingelheim, Daiichi Sankyo, Eisai, Eli Lilly, Janssen Pharmaceutical, Kyowa Pharmaceutical, Lundbeck Japan, Luye Pharma, Mitsubishi Tanabe Pharma, Otsuka Pharmaceutical, Pfizer, Sumitomo Dainippon Pharma, Taisho Toyama Pharma‐ceutical, and Takeda Pharmaceutical.

## Data Availability

All data generated or analyzed during this study are included in this published article. No additional datasets were generated or analyzed.
